# The Relationship Between Internalized Homophobia, Social Anxiety, and Psychological Resilience in Lesbian, Gay and Bisexual Individuals

**DOI:** 10.1192/j.eurpsy.2025.2425

**Published:** 2025-08-26

**Authors:** H. Öztürk, M. Hacıoğlu Yıldırım

**Affiliations:** 1 Psychiatry, Republic of Türkiye Ministry of Health Adana City Training and Research Hospital, Adana; 2 Psychiatry, Trauma and Disaster Study Unit of the Türkiye Psychiatric Association, CETAD trainer and coordinator of the CETAD Trauma Scientific Study Unit, İstanbul, Türkiye

## Abstract

**Introduction:**

Lesbian, gay, and bisexual (LGB) individuals with high psychological resilience are less likely to experience minority stress and mental health issues. This study will investigate the role of psychological resilience in the relationship between internalized homophobia and social anxiety among LGB individuals.

**Objectives:**

LGB individuals experience higher levels of social anxiety compared to heterosexual individuals. This is due to internalized homophobia, a result of minority stress. Psychological resilience is a significant factor that determines LGB individuals’ ability to cope with these challenges. This study aims to determine the levels of internalized homophobia, social anxiety, and psychological resilience in LGB individuals and to examine the relationships between these concepts. Additionally, the study will investigate the mediating role of psychological resilience in the impact of sociodemographic and clinical characteristics of LGB individuals on internalized homophobia and social anxiety.

**Methods:**

The study used a cross-sectional design to survey 131 LGB individuals. Participants were recruited through snowball and purposive sampling. Data was collected using sociodemographic and clinical questionnaires, the Internalized Homophobia Scale, Liebowitz Social Anxiety Scale, and Adult Resilience Scale.

**Results:**

Gay individuals exhibited significantly higher levels of psychological resilience and lower levels of social anxiety compared to lesbian and bisexual individuals. Factors such as younger age, lower socioeconomic status, unemployment, female gender, suicide attempts, seeking psychological support and partial disclosure of sexual orientation were associated with higher levels of social anxiety. A negative correlation was found between levels of social anxiety and psychological resilience. Additionally, a negative correlation was observed between the social resources subscale of psychological resilience and levels of internalized homophobia. Structural style, social resources, and social competence subscales of social anxiety and psychological resilience were found to be significant predictors of internalized homophobia.

**Conclusions:**

This study provides a deeper understanding of the psychological challenges faced by LGB individuals and the underlying factors contributing to these challenges. It particularly focuses on the complex relationships between internalized homophobia, social anxiety, and psychological resilience. Attending to internalized homophobia and social anxiety in the assessment of LGB individuals is crucial for preventing mental health issues and enhancing psychological resilience. These findings may contribute to the development of more effective psychological support programs for LGB individuals.

**Disclosure of Interest:**

None Declared

